# Utilizing Green Design as Workplace Innovation to Relieve Service Employee Stress in the Luxury Hotel Sector

**DOI:** 10.3390/ijerph17124527

**Published:** 2020-06-23

**Authors:** Heesup Han, Antonio Ariza-Montes, Gabriele Giorgi, Soyeun Lee

**Affiliations:** 1 College of Hospitality and Tourism Management, Sejong University, 98 Gunja-Dong, Gwanjin-Gu, Seoul 143-747, Korea; heesup.han@gmail.com; 2 Department of Management, Universidad Loyola Andalucía, C/ Escritor Castilla Aguayo, 4 14004 Cordóba, Spain; ariza@uloyola.es; 3 Department of Human Science, Università Europea di Roma, Via degli Aldobrandeschi, 190 00163 Roma, Italy; gabriele.giorgi@unier.it

**Keywords:** green design as workplace innovation, stress resilience, hotel employees, emotional exhaustion, job satisfaction, job involvement, organizational citizenship behavior

## Abstract

This study is an empirical endeavor to provide a clear comprehension regarding how hotel green design as a workplace innovation contributes to relieving employee stress and emotional fatigue and improves job satisfaction and involvement in the formation of organizational citizenship behavior. A quantitative process was employed to attain the research goal. Our empirical findings demonstrated that a green design as a workplace innovation boosts the stress resilience that leads to the decreased emotional exhaustion and increased job satisfaction. In addition, job satisfaction and job involvement were the crucial drivers of the organizational citizenship behavior among the luxury hotel service employees. Moreover, stress resilience, satisfaction and involvement were significant mediators. Job satisfaction and involvement had the strongest influence on organizational citizenship behavior than other variables. The findings of our research will help hotel proprietors to invent efficient strategies to minimize employee stress and maximize fulfillment at work, which will eventually enhance the organizational citizenship behavior.

## 1. Introduction

Green design—along with the green physical environments—has been a fundamental topic in the diverse business sectors for the past few decades [1,2,3]. The green physical environments of a building—as well known as biophilic atmospherics—are considered as a significant instance of nature-based solutions for occupant mental/emotional health [3,4,5,6]. Indeed, the effective use of the green design of a company can result in decreased anxiety/stress and increased comfort/well-being among the visitors [7,8,9] particularly in the service consumption situation [2,3]. This green design is also vital and effective for workers in the diverse places of hospitality/service firms [10,11,12]. Green design can be an important factor that affects employee behaviors especially in the hotel industry [1].

For hotel entrepreneurs, inducing employee organizational citizenship behavior is essential for the hotel company’s long-term success [13,14,15,16]. When employees are active in organizational citizenship behaviors, they contribute to their organization in a positive way that goes beyond the defined work roles, which is considerably beneficial to any hotel company. Since the green atmospherics of a building are of great importance to trigger occupant positive reactions and attitudes to the company [3,4,11,12,16,17], it is irrefutable that implementing green design in a hotel can be one possible approach to elicit employee organizational citizenship behavior at the hotel.

Despite its importance, the role of a green design to explain the employee responses/behaviors in the hotel industry has not been abundantly researched. Specifically, little empirical research has enriched the body of knowledge regarding a green design as workplace innovation and its effect on the process of generating employee organization citizenship behavior. In addition, even though service employee stress and emotional burnout are becoming an increasing critical concern in the hotel industry, an empirical endeavor of exploring how these factors are linked to a green design have not been clearly unearthed. Moreover, the existing knowledge pertinent to the influence of these types of relationships on job satisfaction, job involvement and organizational citizenship behavior among hotel employees are very limited.

The present research was intended to bridge the gap in existing literature. The objectives of this study were (1) to assess the effect of hotel green design as workplace innovation on the formation of organizational citizenship behavior, (2) to test the intricate associations among green designs, stress resilience, emotional exhaustion, job satisfaction and job involvement, (3) to uncover how these types of relationships contribute to increasing the organizational citizenship behavior, (4) to examine the mediating role of the study variables within the suggested theoretical model, and (5) to explore the comparative criticality in the group of the research constructs in determining the organizational citizenship behavior.

A comprehensive review of the literature will follow next. The research methodology that comprise of the measurement development, the data collecting procedure and the sample characteristics are then provided. Subsequently, the outcomes of measurement and the structural model assessments are presented. Lastly, the implications, the limitations and the future research areas are discussed.

## 2. Review of the Literature

### 2.1. Green Design as Workplace Innovations

Green design is an emerging issue in the business sector that includes hospitality and tourism businesses [3,18]. Recently, green designs have been considered as an innovative tactic that can provide a variety of benefits for occupant health [2,19]. Indeed, many researchers in their recent studies [1,2,20] indicated that the benefits for humans can be decreased stress, increased positive emotions, minimized stress and improved physical activities/health. According to them, the benefits also exist for the environment, which include reduced pollution, decreased noise, improved air freshness and increased water quality. Green décor (the interior and the exterior), green items(potted plants, flowers, green walls and trees), green spaces (green spaces/places for rest/leisure) and natural light (glass ceilings, glass walls and glass windows) are all critical constituents of a green design of a building [2,3,20]. Particularly, these constituents are the critical aspects of sustainable hotel buildings [1,2]. In addition, these types of components of a green design can be crucial factors with service or workplace quality and satisfaction assessment [12].

Prior empirical studies showed that the green design of a building can be one of the efficient ways to make the occupants feel mentally healthy and relieve their stress [2,18]. In hotels, the occupants can be customers as well as the workers [1,2]. As stated by Han et al. [12], the hotel’s eco-friendly and healthy material setting helps people feel the well-being and mental health perception. In a hotel context, Trang et al. [2] also found that green indoor and outdoor atmospherics elicit individual positive responses and behaviors regarding the hotel. Han and Hyun [1] examined occupant behaviors in the hotel sector. Their empirical results revealed that a green physical environment affects stress resilience and emotional burnout. They also identified that the attachments of hotel occupants and other positive behaviors toward the company had a significant impact on the green physical environment. On the basis of this observation, we can propose the following hypothesis.

**Hypothesis** **1:** *Green designs have a positive influence on stress resilience.*

### 2.2. Stress Resilience

Stress resilience is indisputably an imperative issue across the globe, since the individuals who cope with mental health problem have been increasing in a rapid manner for the last few decades [3,4,6,21,22]. Stress resilience refers to one’s self-assessment concerning his/her capability to handle a psychological or mental crisis, which includes stress and anxiety and return to a normal state, such as feeling refreshed [3]. This normal state can be described as a pre-crisis status. His conceptualization of stress resilience is coherent with Han et al.’s [18] description. Han et al. [18] (p. 3) defined stress resilience as “one’s ability to navigate psychological/mental difficulty/adversity in a manner that protects his/her mental health, psychological well-being, and life satisfaction”. According to them, psychological health and well-being along with life satisfaction are the core aspects of stress resilience. In the same manner, Hwang and Lee [22] asserted that one’s stress resilience protects himself/herself from the harmful influence of the potential stressors. The stress resilience of customers and workers are irrefutably a vital issue in the global service business industry [1,6,10].

Evidence in the existing studies indicates that patron- or worker-exposure to green atmospherics brings their psychological comfort and mental well-being [1,3,4,18,22]. In the tourism sector, Han et al. [18] investigated airport user behaviors. They found that user psychological resilience reduces emotional exhaustion and increases their satisfaction with healthy airport atmospherics. Yu [3] examined the customer retention process in the hospitality sector. His empirical finding showed that customer stress resilience increases their emotional comfort and decreases their anxiety. He also identified that stress resilience strengthened the connection between the customers and the brand. In addition, Hwang and Lee [22] demonstrated the essential role of psychological resilience to generate the mental well-being and the service evaluation process. Stress resilience is also crucial for worker behaviors. Indeed, Han and Hyun [1] uncovered that stress resilience contributes to minimizing employee burnout and accelerating their job satisfaction level. Accordingly, the following hypotheses are presented.

**Hypothesis** **2:** *Stress resilience has a negative influence on emotional exhaustion.*

**Hypothesis** **3:** *Stress resilience has a negative influence on job satisfaction.*

### 2.3. Emotional Exhaustion

Because of the uncertainty of the recent service business world, worker emotional exhaustion is increasingly becoming a critical issue [23]. The emotional exhaustion among employees is nowadays a prevalent and well-known phenomenon in the workplace even though how it is formed and how it affects employee functioning are not clearly illuminated [24]. Emotional exhaustion refers to employees’ general feelings of being used-up, worn-out and exhausted of affective/energy resources [23,25]. On the word of David et al. [23], emotional exhaustion is generally considered the main dimension of employee fatigue at work. Their assertion is in line with Tang and Vandenberghe’s [24] indication that emotional exhaustion as a main facet of worker well-being is a core constituent of burnout, such as a feeling of exhaustion at work. Indisputably, emotional exhaustion has devastating impact on worker job performances and behaviors [23,24,25].

For instance, Tang and Vandenberghe [24] explored that individual emotional exhaustion in the workplace reduces job satisfaction and the degree of dedication to the company in organizational behavior sector. Similarly, David et al. [26] identified that emotional exhaustion among workers lowers their job satisfaction and productivity in their empirical research, which poses considerable organization costs. Pietilä et al. [19] investigated a green physical environment and individual’ recreation behaviors. Their findings revealed that green spaces and atmospherics are highly associated with individual stress resilience, and this relationship affects mental health, emotional exhaustion, daily outings, and outdoor recreation visits. In the tourism context, Moon et al. [11] uncovered that individual emotional comfort/well-being derived from the excellent performances of airport green atmospherics help them feel pleased. Their results also showed that this type of positive emotional state leads to increased satisfaction. Given these lines of reasoning, we develop the following hypothesis.

**Hypothesis** **4:** *Emotional exhaustion has a negative influence on job satisfaction.*

### 2.4. Job Satisfaction

Job satisfaction, one of the dimensions of employee’s decision-making process and behavior, is a subject that extensively studied in various sector of organizational behavior [15,27,28]. According to Appiah [28], job satisfaction indicates one’s favorable affective or fulfillment state that is derived from evaluating his/her job experiences, which include experiences at work. This definition is coherent with Oliver’s [13] conceptualization of satisfaction, which satisfaction is an evaluation process of a company and its attribute performances. Since the hospitality industry, which includes the hotel sector, is a labor-intensive service industry, elevating job satisfaction has become increasingly important in eliciting positive responses/behaviors of workers to the company, which involves job attachment/involvement, loyalty to the firm and organizational citizenship behaviors [28,29]. Indeed, Bilgin and Bemirer [27] explored that job satisfaction of hotel employees is significantly linked to their job involvement. In addition, Ilies et al. [15] discovered job satisfaction is a critical aspect to explicate employee attitudes and organizational citizenship behaviors. Given this, the following hypotheses are developed.

**Hypothesis** **5:** *Job satisfaction has a positive influence on job involvement.*

**Hypothesis** **6:** *Job satisfaction has a positive influence on organizational citizenship behaviors.*

### 2.5. Job Involvement

Job involvement refers to their dynamic participation in their job. Job involvement is a significant constituent of workers’ positive responses/behaviors to for the organization [30] and is also a deeply absorbed state of workers’ overall job at work [31]. Therefore, the concept is alternatively used with the terms, such as job attachment and job engrossment. A highly job-involved worker is more loyal to his/her company and is engrossed in what he/she does at work [30,32]. In addition, employees with high job involvement levels believe that they are part of the organization and invest considerable efforts to achieve organizational objectives and actively practice citizenship behaviors for the organization [14,33]. The existing studies indicated that job involvement is strongly associated with job satisfaction, commitment and organizational citizenship behavior [14,16,30,33]. Taking these into account, the following hypothesis is developed:

**Hypothesis** **7:** *Job involvement has a positive influence on organizational citizenship behavior.*

### 2.6. Proposed Model

**Figure 1:**
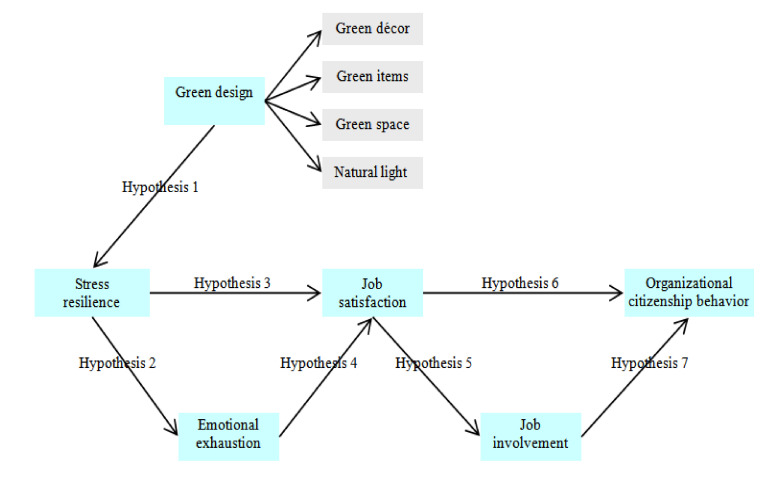
Proposed model and the research hypotheses.

Figure 1 shows our proposed model that is developed to explicate the process of generating organization citizenship behavior in the luxury hotel sector. The proposed theoretical framework encompasses the six research constructs and includes seven research hypotheses that link the variables.

## 3. Methods

### 3.1. Survey Questionnaire Development

For the evaluation of the study constructs, we employed the measurement items from the current literatures [5,19,20,21,31,34,35,36,37,38]. Specifically, we used four items (green décor, green items, green space and natural light) to evaluate a green design (e.g., this hotel has diverse green interior decorations). The four items were used to measure stress resilience (e.g., working at this hotel helps me turn any worry/anxiety into confidence). To evaluate the emotional exhaustion, three items were utilized (e.g., I feel used up at the end of the workday). We measured job satisfaction using three items (e.g., I feel satisfied with my overall job). Lastly, the organizational citizenship behavior was assessed using three items (e.g., I help my coworkers whenever possible). All the items were assessed using a seven Likert’s scale that ranged from 1—strongly agree to 7—strongly disagree. A pretest was performed by hotel industry professionals and academics. The questionnaire was improved based on their comments. The questionnaire was then finalized after a thorough review by the hotel academic experts and a slight amendment.

### 3.2. The Data Collection Procedure

To collect data, we used a survey system from an online research firm. A survey invitation, which included the URL and the research description, was sent to the employees in eleven luxury hotels located in South Korea. The employees were from diverse guest-contact departments of a hotel, which included the guestroom, the food and beverage, the concierge and the promotion department. The participants accessed the questionnaire by clicking the link and asked to read the introductory letter thoroughly and then filled in the questionnaire. The average completion time for the survey was 10.5 minutes. In total 280 valid samples were collected and utilized for empirical analysis.

### 3.3. Demographic Profiles of the Samples

From the 280 responses, 56.8% were female hoteliers and 43.2% were male hoteliers. Further, 45.4% of respondents stated their age was 31–40 years old, which was followed by less than 30 years old (32.9%), between 41–50 years old (13.9%), and more than 51 years old (7.9%). In addition, about 42.9% of the respondents indicated that their income was $30,000 or less, which was followed by between $30,001–$50,000 (41.1%) and $50,001 or more (16.1%). In regard to their education level, about 43.3% of the participants were those with a university degree, followed by 2-year college degree (34.3%), graduate degree (20.4%) and high school degree (1.4%). In addition to this, about 29.6% of the participants reported being employed between 1–3 years, which was followed by 10 years or more (29.3%), between 4–6 years and between 7–9 years (18.9%).

## 4. Analysis and Results

### 4.1. Measurement Model Evaluation

The SPSS 20 (IBM, New York, NY, USA) and AMOS 20 (IBM, New York, NY, USA) were used for data analysis. The quality of the multiple measurement items was assessed by conducting confirmatory factor analysis (CFA). The measurement model had a good fit with the data (χ^2^ = 368.099, *df* = 152, χ^2^/*df* = 2.422, *p* < 0.001, RMSEA = 0.071, CFI = 0.957, IFI = 0.957 and TLI = 0.946). First, reliability was assessed by composite reliability (CR). As seen in Table 1, all value of composite reliability (CR) exceeded the threshold of 0.70 [39], demonstrating an internal consistency among the measures within each construct (green design = 0.919, stress resilience = 0.946, emotional exhaustion = 0.893, job satisfaction = 0.764, job involvement = 0.964 and organizational citizenship behavior = 0.870). Next, convergent validity was evident by the average variance extracted values (AVE) that all construct AVEs were greater than the suggested threshold of 0.50 [39] (green design = 0.741, stress resilience = 0.814, emotional exhaustion = 0.739, job satisfaction = 0.524, job involvement = 0.898 and organizational citizenship behavior = 0.692). Last, all AVE values were greater than the squared correlations coefficients between the research constructs. Thus, discriminant validity of the constructs was also supported.

### 4.2. Structural Equation Modeling and the Hypotheses Test

**Figure 2:**
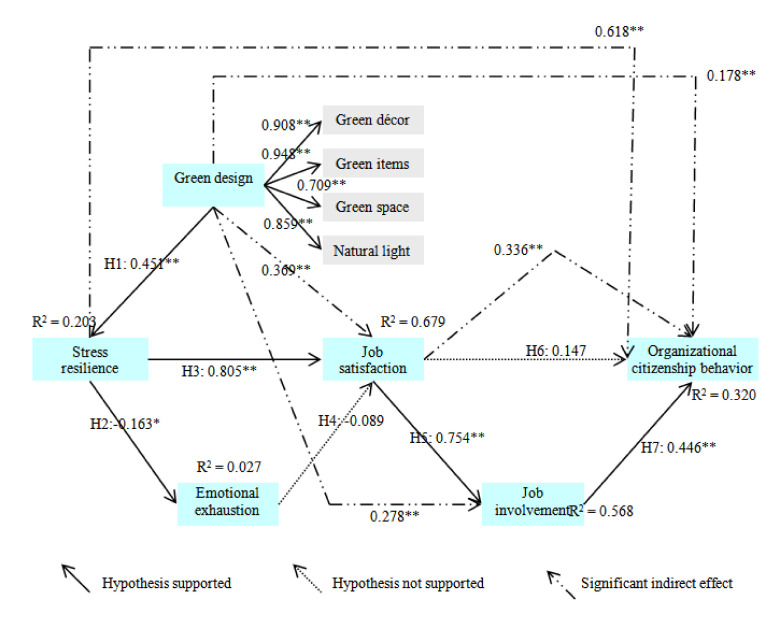
Results of the structural model and the baseline model assessment (*n* = 280).

Structural equation modeling (SEM) was developed to test hypothesized relationship. The overall fit of our conceptual model met an adequate level (χ^2^ = 441.719, *df* = 160, *p* < 0.001, χ^2^/*df* = 2.761, RMSEA = 0.079, CFI = 0.944, IFI = 0.944 and TLI = 0.933). Table 2 and Figure 2 contain the detailed results of the SEM assessment. The structural model explained about 32.0% of the total variance in the organizational citizenship behavior. It also accounted for approximately 56.8% and 67.9% of the variance in job involvement and job satisfaction, respectively. Moreover, the model explained for about 20.3% of the variance in the stress resilience.

The hypothesized linkage between the green design and the stress resilience was tested. Our result revealed that the green design had a significant effect on the stress resilience (β = 0.451 and *p* < 0.01), thus supporting hypothesis 1. Stress resilience was found to exert a significant effect on emotional exhaustion (β = −0.163 and *p* < 0.05) and job satisfaction (β = 0.805 and *p* < 0.01). Therefore, Hypotheses 2 and 3 were also supported. The emotional exhaustion was not found to significantly affect job satisfaction (β = −0.089 and *p* > 0.05), thus rejecting hypothesis 4. Job satisfaction was found to significantly affect job involvement (β = 0.754 and *p* < 0.01). Yet, its effect on organizational citizenship behavior (β = 0.147 and *p* > 0.05) was not significant. Hence, hypothesis 5 was supported, but hypothesis 6 was rejected. Lastly, job satisfaction was found to have a positive effect on organizational citizenship behavior, thus supporting the hypothesis.

### 4.3. Indirect and Total Effect Assessment

We investigated the indirect effect of research constructs. As Table 3 and Figure 2 show, the green design included a significant indirect impact on job satisfaction (β = 369 and *p* < 0.01), job involvement (β = 0.278 and *p* < 0.01) and organizational citizenship behavior (β = 0.178 and *p* < 0.01). The stress resilience also contained a significant indirect effect on organizational citizenship behavior (β = 0.618 and *p* < 0.01). In addition, job satisfaction had a significant indirect impact on organizational citizenship behavior (β = 0.336 and *p* < 0.01). This finding indicated that stress resilience, job satisfaction, and job involvement acted an essential part as mediators within the suggested framework. Subsequently, we examined the total influence of the research variables and job satisfaction was found to contain the greatest effect on organizational citizenship behavior (β = 0.483 and *p* < 0.01), which was followed by job involvement (β = 0.446 and *p* < 0.01), stress resilience (β = 0.396 and *p* < 0.01) and green design (β = 0.178 and *p* < 0.01). This result demonstrated the relative importance of job satisfaction and job involvement within the proposed theoretical framework.

## 5. Discussion and Implication

Our theoretical framework provided a clear understanding of a luxury hotel employee organization citizenship behavior through a quantitative approach. The proposed model, which was empirically evaluated by the data quality testing and structural equation modeling, and it successfully took the effect of a green design as workplace innovation into account. Based on our evaluation of explanatory power, the model was competent to anticipate the job involvement and predict the organization citizenship behavior. The proposed framework showed how a green design increases the stress resilience, decreases the emotional exhaustion—and how these types of relationships drive job satisfaction, job involvement and citizenship behavior. The constructs within the projected theoretical model satisfactorily explained the variance in job satisfaction, job involvement and citizenship behavior. The effectiveness of our conceptual framework for the apparent understanding of the organizational citizenship behavior formation among the luxury hotel service employees was evident given this.

The key aspects of our research model were cognitive (stress resilience), affective (emotional exhaustion and job satisfaction), conative (job involvement) and environmental (green design) dimensions, which in conjunction explicate the organizational citizenship behavior. As previously earlier, the competence of the model was demonstrated in an empirical manner. Our research framework also encompassed a mediation mechanism, and it therefore deepened the cognitive, affective and conative associations successfully. In the highly competitive marketplace across the globe, knowing employee positive decision/behaviors for a company is inevitable for the survival/success of any hotel. This research successfully enriched the extant literature further, which will help the proprietors clearly illuminate service employees’ convoluted decision formation and the organizational citizenship behavior of the hotel that they work for.

Our investigation of the relative importance of the research variables demonstrated that job satisfaction and job involvement better contribute to inducing employee organizational citizenship behavior than other research constructs. In particular, these factors acted as prominent determinants of organizational citizenship behavior in the group of luxury hotel staffs. This result supported the findings in the previous studies [28,30,33], which showed the significance of satisfaction and involvement in individual behaviors in the tourism literature. Moreover, the results of this study confirmed the premise of the need to incorporate the satisfaction evaluation and the involvement processes into the research framework for the employees’ organizational behavior. The present research included the salient dimensions to the formation of organization citizenship behavior in a successful manner, which was lacking in the theoretical frameworks of the existing studies. Dealing with job satisfaction and job involvement can be a fundamental approach to boost the hotel employees’ organizational citizenship activities given this.

Our result indicated that a green design is a crucial factor to improve stress resilience. This finding implies that implementing a green design in a hotel is important to help the hotel employees feel refreshed, reduce anxiety and increase their confidence levels. Based on our results, a green design also contributes to inducing job satisfaction, job involvement and organizational citizenship behavior. The present research and its results about the effect of a green design is theoretically meaningful, because it is one of very few studies that empirically demonstrated the value of a green design to elicit hotel employee positive responses and behaviors through the illumination of its significant linkage with stress resilience. From a managerial perspective, hotel managers should improve green décor, increase plants/flowers/trees, increase green spaces and enlarge glass ceilings/walls/windows. This effort would be an essential tactic to efficiently deal with employee stress resilience, satisfaction, involvement and citizenship behavior for their organization.

Our finding indicated the significant mediating role of stress resilience, job satisfaction and job involvement within the hypothesized theoretical framework. These results are consistent with prior studies highlighting the importance of these constructs as mediators [37,40,41]. Our results imply that stress resilience, job satisfaction and job involvement acted as vital factors, because they maximized the effect of their antecedents on their outcome factors. Theoretically, the proposed framework helped us elucidate the role of the critical distal predictors of organizational citizenship behavior. Recognizing the crucial contribution of stress resilience, job satisfaction and job involvement, hotel proprietors should deal with the mediation factors to take full advantage of the influence of hotel green design to escalate the organizational citizenship behavior that eventually results in the successful operation of the hotels.

The present study contains a few limitations. The first limitation involves generalizability. Since this study is centered on the luxury hotel sector, generalizing our results to employee behaviors in other tourism/business sectors, which include cruises, airports and restaurants, need to be done with some caution. Replication of this study is necessary for future studies with different contexts. Second, despite its strong prediction power for the organizational citizenship behavior, our conceptual framework needs to be further fortified by integrating additional constructs for its completeness. The extension effort of the proposed framework for future research could lead to filling the possible omissions associated with the theorization made in the present research.

## 6. Conclusions

The present research successfully developed a robust framework of the luxury hotel service employees’ organizational citizenship behavior by exploring the complicated relationships among a green design, stress resilience, emotional exhaustion, job satisfaction and job involvement. This study was the first to empirically uncover the magnitude of the influence of a green design as a workplace innovation on the elicitation process of the organizational citizenship behavior. We also believe that this study contributes to development of the current literature on what factors are under the influence of a green design and how these factors are effectively interrelated with each other within the projected hypothetical model. Building on the extant literature, the current study includes vital theoretical and managerial meanings because it provides a deeper comprehension of the possible outcomes of hotel green design, which reduce psychological stress and emotional exhaustion, enhance job satisfaction and job involvement and increase organizational citizenship behavior.

## Figures and Tables

**Table 1:** Measurement model estimation results (*n* = 280).

Variables.	(a)	(b)	(c)	(d)	(e)	(f)	Mean (SD)	CR	AVE
(a) Green design	1.000	–	–	–	–	–	5.338 (1.326)	0.919	0.741
(b) Stress resilience	0.422 ^a^ (0.178) ^b^	1.000	–	–	–	–	4.486 (1.476)	0.946	0.814
(c) Emotional exhaustion	0.156 (0.024)	−0.171 (0.029)	1.000	–	–	–	4.454 (1.530)	0.893	0.739
(d) Job satisfaction	0.236 (0.056)	0.603 (0.364)	−0.182 (0.033)	1.000	–	–	3.810 (1.455)	0.764	0.524
(e) Job involvement	0.367 (0.135)	0.636 (0.404)	−0.177 (0.031)	0.572 (0.327)	1.000	–	4.866 (1.533)	0.964	0.898
(f) Organizational citizenship behavior	0.462 (0.213)	0.351 (0.123)	−0.032 (0.001)	0.285 (0.081)	0.500 (0.025)	1.000	5.986 (.938)	0.870	0.692

Note: Goodness of fit statistics for the measurement model: χ^2^ = 368.099, *df* = 152, χ^2^/*df* = 2.422, *p* < 0.001, ^a^ Correlations between the variables are below the diagonal. ^b^ The squared correlations are within the parentheses. SD: Standard Deviation; CR: Composite Reliability; AVE: Average Variance Extracted values.

**Table 2:** Structural model estimation results (*n* = 280).

Proposed Paths				β	*t*-Values
H1	Green design	→	Stress resilience	0.451	7.463 **
H2	Stress resilience	→	Emotional exhaustion	−0.163	−2.568 *
H3	Stress resilience	→	Job satisfaction	0.805	9.979 **
H4	Emotional exhaustion	→	Job satisfaction	−0.089	−1.784
H5	Job satisfaction	→	Job involvement	0.754	9.484 **
H6	Job satisfaction	→	Organizational citizenship behavior	0.147	1.437
H7	Job involvement	→	Organizational citizenship behavior	0.446	4.323 **
Total variance explained: R^2^ for organizational citizenship behavior = 0.320 R^2^ for job involvement = 0.568 R^2^ for job satisfaction = 0.679 R^2^ for emotional exhaustion = 0.027 R^2^ for stress resilience = 0.203					

Note: Goodness of fit statistics for the structural model: χ^2^ = 441.719, *df* = 160, *p* < 0.001, χ^2^/*df* = 2.761, RMSEA = 0.079, CFI = 0.944, IFI = 0.944 and TLI = 0.933; * *p* < 0.05 and ** *p* < 0.01.

**Table 3:** Indirect impact and total impact assessment (*n* = 280).

On	Indirect Effects of				
	Green Design	Stress Resilience	Emotional Exhaustion	Job Satisfaction	Job Involvement
Emotional exhaustion	−0.074	–	–	–	–
Job satisfaction	0.369 **	–	–	–	–
Job involvement	0.278 **	0.015	−0.067	–	–
Organizational citizenship behavior.	0.178 **	0.618 **	−0.043	0.336 **	–
Total impact on organizational citizenship behavior: β for job involvement = 0.446 ** β for job satisfaction = 0.483 ** β for emotional exhaustion = −0.043 β for stress resilience = 0.396 ** β for green design = 0.178 **					

Note: Goodness of fit statistics for the structural model: χ^2^ = 441.719, *df* = 160, *p* < 0.001, χ^2^/*df* = 2.761, RMSEA = 0.079, CFI = 0.944, IFI = 0.944 and TLI = 0.933; * *p* < 0.05 and ** *p* < 0.01.
